# 5-Meth­oxy-2-{[4-(morpholin-4-yl)phen­yl]imino­meth­yl}phenol

**DOI:** 10.1107/S1600536811034659

**Published:** 2011-08-27

**Authors:** K. Dhahagani, K. Manvizhi, G. Chakkaravarthi, G. Anbalagan, G. Rajagopal

**Affiliations:** a Department of Chemistry, Government Arts College, Melur 625 106, India; b Department of Chemistry, Anand Institute of Higher Technology, Kazhipattur, Chennai 603 103, India; c Department of Physics, CPCL Polytechnic College, Chennai 600 068, India; d Department of Physics, Presidency College (Autonomous), Chennai 600 005, India

## Abstract

In the title compound, C_18_H_20_N_2_O_3_, the dihedral angle between the two aromatic rings is 33.66 (6)°. The morpholine ring adopts a chair conformation. The mol­ecular structure is stabilized by an intra­molecular O—H⋯N hydrogen bond. In the crystal, mol­ecules are linked *via* weak inter­molecular C—H⋯O and C—H⋯π inter­actions.

## Related literature

For the biological activity of morpholine derivatives, see: Lan *et al.* (2010 ▶); Raparti *et al.* (2009 ▶). For standard bond lengths, see: Allen *et al.* (1987 ▶). For a related structure, see: Yang *et al.* (2011 ▶). For the definition of puckering parameters, see: Cremer & Pople (1975 ▶). For graph-set notation, see: Etter *et al.* (1990 ▶).
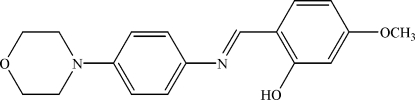


## Experimental

### 

#### Crystal data

C_18_H_20_N_2_O_3_
*M* *_r_* = 312.36
Monoclinic, 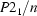

*a* = 10.623 (6) Å
*b* = 9.106 (5) Å
*c* = 16.640 (5) Å
β = 97.446 (6)°
*V* = 1596.2 (13) Å^3^
*Z* = 4
Mo *K*α radiation
μ = 0.09 mm^−1^
*T* = 295 K
0.28 × 0.24 × 0.20 mm

#### Data collection

Bruker Kappa APEXII diffractometer
Absorption correction: multi-scan (*SADABS*; Sheldrick, 1996 ▶) *T*_min_ = 0.975, *T*_max_ = 0.982
21296 measured reflections
4941 independent reflections
2761 reflections with *I* > 2σ(*I*)
*R*_int_ = 0.031
Standard reflections: 0

#### Refinement

*R*[*F* ^2^ > 2σ(*F* ^2^)] = 0.066
*wR*(*F* ^2^) = 0.204
*S* = 1.05
4941 reflections
210 parameters
H-atom parameters constrained
Δρ_max_ = 0.29 e Å^−3^
Δρ_min_ = −0.27 e Å^−3^

### 

Data collection: *APEX2* (Bruker, 2004 ▶); cell refinement: *SAINT* (Bruker, 2004 ▶); data reduction: *SAINT*; program(s) used to solve structure: *SHELXS97* (Sheldrick, 2008 ▶); program(s) used to refine structure: *SHELXL97* (Sheldrick, 2008 ▶); molecular graphics: *PLATON* (Spek, 2009 ▶); software used to prepare material for publication: *SHELXL97*.

## Supplementary Material

Crystal structure: contains datablock(s) global, I. DOI: 10.1107/S1600536811034659/bt5628sup1.cif

Structure factors: contains datablock(s) I. DOI: 10.1107/S1600536811034659/bt5628Isup2.hkl

Supplementary material file. DOI: 10.1107/S1600536811034659/bt5628Isup3.cml

Additional supplementary materials:  crystallographic information; 3D view; checkCIF report

## Figures and Tables

**Table 1:** Hydrogen-bond geometry (Å, °) *Cg*2 and *Cg*3 are the centroids of the C5–C10  and C12–C17 rings, respectively.

*D*—H⋯*A*	*D*—H	H⋯*A*	*D*⋯*A*	*D*—H⋯*A*
O2—H2⋯N2	0.82	1.87	2.600 (2)	148
C14—H14⋯O3^i^	0.93	2.53	3.412 (3)	158
C18—H18*B*⋯O1^ii^	0.96	2.44	3.289 (3)	148
C2—H2*A*⋯*Cg*3^iii^	0.97	2.92	3.799 (4)	152
C16—H16⋯*Cg*2^iv^	0.93	2.86	3.663 (3)	146

Symmetry codes: (i) 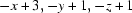; (ii) 
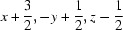
; (iii) 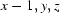; (iv) 
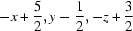
.

**Fig. 1.**
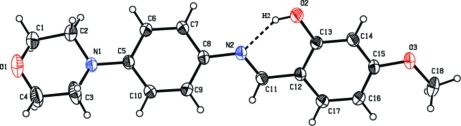
The molecular structure of (I), with atom labels and 30% probability displacement ellipsoids for non-H atoms.

**Fig. 2.**
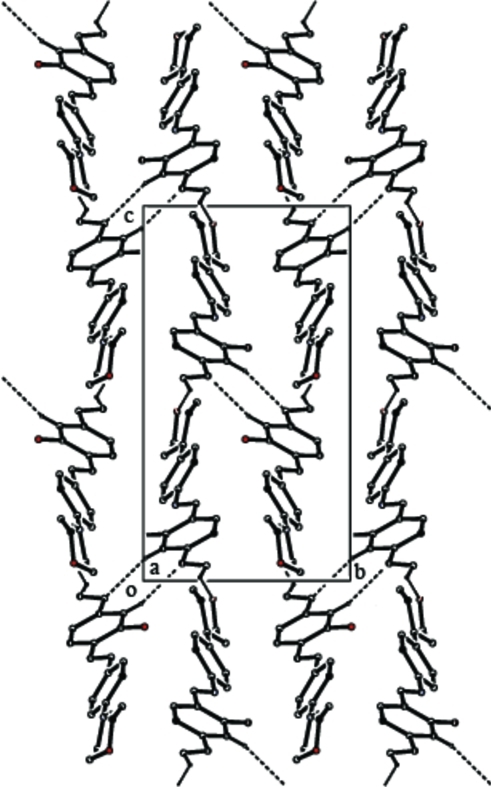
The packing of (I), viewed down a axis. Intermolecular Hydrogen bonds are shown as dashed lines. H atoms not involved in hydrogen bonding have been omitted.

## supplementary crystallographic information

### Comment

Morpholine derivatives possess anticancer and antimicrobial (Lan *et al.*, 2010; Raparti *et al.*, 2009) activities. The goemetric parameters of the title compound (I) are comparable with the literature values and reported related structure (Allen *et al.*, 1987; Yang *et al.*, 2011).

The mean planes of the two benzene rings (C5-C10) and (C12-C17) are oriented at an angle of 33.66 (6)°. The morpholine ring adopts chair conformation [Puckering parameters are Q = 0.470 (3)Å, θ = 7.0 (2)° and Φ = 11 (3)° (Cremer & Pople, 1975) for the ring (O1/C1/C2/N1/C3/C4)].

The molecular structure is stabilized by weak intramolecular O—H···N hydrogen bonding. In the crystal structure, the molecules are linked via weak intermolecular C—H···O and  C—H···π (Fig. 2 and Table 1) interactions. Intramolecular O2-H2···N2 hydrogen bonding generates a six-membered ring, with S(6) graph-set motif and the intermolecular C13-H13···O3 interaction generates an eight-membered ring, with  R_2_^2^(8) graph-set motif.

### Experimental

An ethanolic solution (20 ml) of  4-(4-aminophenyl)morpholine (10 mmol) was magnetically stirred in a round bottom flask followed by drop wise addition of ethanolic solution of 4-methoxysalicylaldehyde (10 mmol). The reaction mixture was then refluxed for two hours and upon cooling to 273 K, a   pale yellow crystalline solid precipitates from the mixture. The solid which is separated out was filtered washed with ice cold ethanol and dried in vaccuo over anhydrous CaCl_2_. Single crystals suitable for the X-ray diffraction were obtained by slow evaporation of a solution of the title compound in methanol at room temperature. Melting Point: 457 K.

### Refinement

All H atoms were positioned geometrically with C—H = 0.93–0.97 Å and O–H = 0.82 Å and allowed to ride on their parent atoms, with Uiso(H) = 1.5 Ueq(O) and 1.2 Ueq(C).

### Crystal data

**Table:**

C_18_H_20_N_2_O_3_	*F*(000) = 664
*M**_r_* = 312.36	*D*_x_ = 1.300 Mg m^−^^3^
Monoclinic, *P*2_1_/*n*	Mo *K*α radiation, λ = 0.71073 Å
Hall symbol: -P 2yn	Cell parameters from 4380 reflections
*a* = 10.623 (6) Å	θ = 2.5–30.6°
*b* = 9.106 (5) Å	µ = 0.09 mm^−^^1^
*c* = 16.640 (5) Å	*T* = 295 K
β = 97.446 (6)°	Block, colourless
*V* = 1596.2 (13) Å^3^	0.28 × 0.24 × 0.20 mm
*Z* = 4	

### Data collection

**Table:**

Bruker Kappa APEXII diffractometer	4941 independent reflections
Radiation source: fine-focus sealed tube	2761 reflections with *I* > 2σ(*I*)
graphite	*R*_int_ = 0.031
ω and φ scans	θ_max_ = 30.7°, θ_min_ = 2.2°
Absorption correction: multi-scan (*SADABS*; Sheldrick, 1996)	*h* = −9→15
*T*_min_ = 0.975, *T*_max_ = 0.982	*k* = −12→12
21296 measured reflections	*l* = −23→23

### Refinement

**Table:**

Refinement on *F*^2^	Primary atom site location: structure-invariant direct methods
Least-squares matrix: full	Secondary atom site location: difference Fourier map
*R*[*F*^2^ > 2σ(*F*^2^)] = 0.066	Hydrogen site location: inferred from neighbouring sites
*wR*(*F*^2^) = 0.204	H-atom parameters constrained
*S* = 1.05	*w* = 1/[σ^2^(*F*_o_^2^) + (0.089*P*)^2^ + 0.3452*P*] where *P* = (*F*_o_^2^ + 2*F*_c_^2^)/3
4941 reflections	(Δ/σ)_max_ < 0.001
210 parameters	Δρ_max_ = 0.29 e Å^−^^3^
0 restraints	Δρ_min_ = −0.27 e Å^−^^3^

### Fractional atomic coordinates and isotropic or equivalent isotropic displacement parameters (Å^2^)

**Table:**

	*x*	*y*	*z*	*U*_iso_*/*U*_eq_	
C1	0.4258 (2)	0.3507 (4)	0.86902 (17)	0.0858 (8)	
H1A	0.3592	0.4221	0.8539	0.103*	
H1B	0.3974	0.2575	0.8448	0.103*	
C2	0.5421 (2)	0.3978 (3)	0.83441 (17)	0.0756 (7)	
H2A	0.5265	0.3921	0.7758	0.091*	
H2B	0.5609	0.4993	0.8491	0.091*	
C3	0.6624 (2)	0.2790 (4)	0.94823 (13)	0.0993 (11)	
H3A	0.6977	0.3649	0.9774	0.119*	
H3B	0.7214	0.1985	0.9605	0.119*	
C4	0.5395 (2)	0.2410 (4)	0.97652 (15)	0.1022 (11)	
H4A	0.5149	0.1435	0.9570	0.123*	
H4B	0.5519	0.2374	1.0353	0.123*	
C5	0.75890 (16)	0.31894 (19)	0.82520 (9)	0.0409 (4)	
C6	0.76084 (19)	0.3993 (2)	0.75393 (12)	0.0559 (5)	
H6	0.6885	0.4501	0.7320	0.067*	
C7	0.86803 (19)	0.4047 (2)	0.71547 (11)	0.0549 (5)	
H7	0.8667	0.4599	0.6683	0.066*	
C8	0.97710 (16)	0.33047 (18)	0.74518 (9)	0.0396 (4)	
C9	0.97771 (17)	0.2539 (2)	0.81688 (10)	0.0472 (4)	
H9	1.0510	0.2051	0.8390	0.057*	
C10	0.87091 (18)	0.2490 (2)	0.85609 (10)	0.0482 (4)	
H10	0.8741	0.1974	0.9045	0.058*	
C11	1.16925 (16)	0.24530 (19)	0.70644 (10)	0.0421 (4)	
H11	1.1595	0.1603	0.7360	0.051*	
C12	1.28060 (16)	0.26119 (18)	0.66578 (9)	0.0393 (4)	
C13	1.30539 (17)	0.39126 (18)	0.62486 (10)	0.0402 (4)	
C14	1.41223 (17)	0.40335 (19)	0.58674 (10)	0.0450 (4)	
H14	1.4284	0.4902	0.5605	0.054*	
C15	1.49540 (17)	0.2874 (2)	0.58729 (10)	0.0461 (4)	
C16	1.47404 (19)	0.1582 (2)	0.62718 (13)	0.0559 (5)	
H16	1.5307	0.0802	0.6280	0.067*	
C17	1.36751 (19)	0.1477 (2)	0.66548 (12)	0.0532 (5)	
H17	1.3530	0.0609	0.6923	0.064*	
C18	1.6838 (2)	0.1948 (3)	0.54114 (18)	0.0868 (8)	
H18A	1.7226	0.1680	0.5944	0.130*	
H18B	1.7482	0.2261	0.5094	0.130*	
H18C	1.6397	0.1117	0.5157	0.130*	
N1	0.64900 (14)	0.30818 (19)	0.86311 (8)	0.0491 (4)	
N2	1.08397 (14)	0.34434 (16)	0.70305 (8)	0.0420 (3)	
O1	0.44192 (16)	0.3353 (2)	0.95242 (11)	0.0846 (6)	
O2	1.22521 (14)	0.50605 (14)	0.62211 (9)	0.0607 (4)	
H2	1.1635	0.4839	0.6445	0.091*	
O3	1.59655 (14)	0.31149 (17)	0.54691 (9)	0.0650 (4)	

### Atomic displacement parameters (Å^2^)

**Table:**

	*U*^11^	*U*^22^	*U*^33^	*U*^12^	*U*^13^	*U*^23^
C1	0.0467 (13)	0.118 (2)	0.0978 (19)	0.0095 (14)	0.0282 (13)	0.0384 (16)
C2	0.0460 (12)	0.0875 (17)	0.0984 (18)	0.0089 (12)	0.0296 (12)	0.0292 (14)
C3	0.0464 (13)	0.211 (4)	0.0423 (11)	−0.0168 (18)	0.0123 (9)	0.0158 (15)
C4	0.0513 (14)	0.204 (4)	0.0542 (13)	0.0027 (19)	0.0187 (11)	0.0345 (17)
C5	0.0358 (9)	0.0504 (10)	0.0378 (8)	−0.0008 (7)	0.0096 (7)	0.0001 (7)
C6	0.0391 (10)	0.0789 (14)	0.0515 (10)	0.0145 (9)	0.0124 (8)	0.0206 (9)
C7	0.0457 (11)	0.0742 (13)	0.0474 (9)	0.0086 (9)	0.0165 (8)	0.0204 (9)
C8	0.0379 (9)	0.0430 (9)	0.0400 (8)	−0.0018 (7)	0.0131 (7)	−0.0021 (6)
C9	0.0398 (9)	0.0576 (11)	0.0457 (9)	0.0105 (8)	0.0118 (7)	0.0082 (8)
C10	0.0458 (10)	0.0589 (11)	0.0422 (8)	0.0064 (9)	0.0142 (8)	0.0119 (8)
C11	0.0431 (10)	0.0409 (9)	0.0443 (8)	−0.0020 (8)	0.0131 (7)	0.0012 (7)
C12	0.0381 (9)	0.0408 (9)	0.0407 (8)	0.0013 (7)	0.0119 (7)	0.0012 (6)
C13	0.0420 (10)	0.0385 (8)	0.0415 (8)	0.0030 (7)	0.0112 (7)	−0.0010 (6)
C14	0.0479 (11)	0.0416 (9)	0.0483 (9)	−0.0017 (8)	0.0169 (8)	0.0034 (7)
C15	0.0383 (9)	0.0576 (11)	0.0454 (9)	0.0020 (8)	0.0161 (7)	0.0031 (8)
C16	0.0473 (11)	0.0549 (11)	0.0693 (12)	0.0179 (9)	0.0218 (9)	0.0137 (9)
C17	0.0519 (11)	0.0478 (10)	0.0640 (11)	0.0099 (9)	0.0228 (9)	0.0160 (8)
C18	0.0587 (15)	0.106 (2)	0.105 (2)	0.0246 (14)	0.0441 (14)	0.0078 (16)
N1	0.0366 (8)	0.0714 (11)	0.0413 (7)	0.0044 (7)	0.0120 (6)	0.0086 (7)
N2	0.0387 (8)	0.0472 (8)	0.0425 (7)	0.0013 (6)	0.0141 (6)	−0.0004 (6)
O1	0.0549 (10)	0.1205 (15)	0.0859 (12)	−0.0139 (10)	0.0373 (9)	−0.0139 (10)
O2	0.0624 (9)	0.0448 (7)	0.0820 (10)	0.0152 (7)	0.0362 (8)	0.0122 (6)
O3	0.0515 (8)	0.0765 (10)	0.0743 (9)	0.0092 (7)	0.0351 (7)	0.0138 (7)

### Geometric parameters (Å, °)

**Table:**

C1—O1	1.383 (3)	C9—C10	1.381 (2)
C1—C2	1.492 (3)	C9—H9	0.9300
C1—H1A	0.9700	C10—H10	0.9300
C1—H1B	0.9700	C11—N2	1.274 (2)
C2—N1	1.430 (3)	C11—C12	1.444 (2)
C2—H2A	0.9700	C11—H11	0.9300
C2—H2B	0.9700	C12—C17	1.386 (2)
C3—N1	1.430 (3)	C12—C13	1.408 (2)
C3—C4	1.485 (3)	C13—O2	1.345 (2)
C3—H3A	0.9700	C13—C14	1.374 (2)
C3—H3B	0.9700	C14—C15	1.376 (3)
C4—O1	1.365 (3)	C14—H14	0.9300
C4—H4A	0.9700	C15—O3	1.357 (2)
C4—H4B	0.9700	C15—C16	1.384 (3)
C5—C10	1.388 (2)	C16—C17	1.372 (3)
C5—C6	1.396 (2)	C16—H16	0.9300
C5—N1	1.400 (2)	C17—H17	0.9300
C6—C7	1.378 (3)	C18—O3	1.421 (3)
C6—H6	0.9300	C18—H18A	0.9600
C7—C8	1.377 (3)	C18—H18B	0.9600
C7—H7	0.9300	C18—H18C	0.9600
C8—C9	1.381 (2)	O2—H2	0.8200
C8—N2	1.415 (2)		
			
O1—C1—C2	114.5 (2)	C8—C9—H9	119.6
O1—C1—H1A	108.6	C9—C10—C5	121.83 (16)
C2—C1—H1A	108.6	C9—C10—H10	119.1
O1—C1—H1B	108.6	C5—C10—H10	119.1
C2—C1—H1B	108.6	N2—C11—C12	121.94 (16)
H1A—C1—H1B	107.6	N2—C11—H11	119.0
N1—C2—C1	111.6 (2)	C12—C11—H11	119.0
N1—C2—H2A	109.3	C17—C12—C13	117.33 (16)
C1—C2—H2A	109.3	C17—C12—C11	120.88 (16)
N1—C2—H2B	109.3	C13—C12—C11	121.79 (15)
C1—C2—H2B	109.3	O2—C13—C14	118.62 (15)
H2A—C2—H2B	108.0	O2—C13—C12	120.85 (16)
N1—C3—C4	112.25 (18)	C14—C13—C12	120.53 (15)
N1—C3—H3A	109.2	C13—C14—C15	120.22 (16)
C4—C3—H3A	109.2	C13—C14—H14	119.9
N1—C3—H3B	109.2	C15—C14—H14	119.9
C4—C3—H3B	109.2	O3—C15—C14	114.92 (16)
H3A—C3—H3B	107.9	O3—C15—C16	124.38 (17)
O1—C4—C3	115.2 (3)	C14—C15—C16	120.70 (17)
O1—C4—H4A	108.5	C17—C16—C15	118.59 (17)
C3—C4—H4A	108.5	C17—C16—H16	120.7
O1—C4—H4B	108.5	C15—C16—H16	120.7
C3—C4—H4B	108.5	C16—C17—C12	122.63 (17)
H4A—C4—H4B	107.5	C16—C17—H17	118.7
C10—C5—C6	116.66 (16)	C12—C17—H17	118.7
C10—C5—N1	121.72 (15)	O3—C18—H18A	109.5
C6—C5—N1	121.62 (16)	O3—C18—H18B	109.5
C7—C6—C5	121.18 (17)	H18A—C18—H18B	109.5
C7—C6—H6	119.4	O3—C18—H18C	109.5
C5—C6—H6	119.4	H18A—C18—H18C	109.5
C8—C7—C6	121.55 (17)	H18B—C18—H18C	109.5
C8—C7—H7	119.2	C5—N1—C2	118.82 (16)
C6—C7—H7	119.2	C5—N1—C3	118.53 (16)
C7—C8—C9	117.88 (16)	C2—N1—C3	114.18 (19)
C7—C8—N2	118.04 (15)	C11—N2—C8	121.75 (15)
C9—C8—N2	123.97 (16)	C4—O1—C1	110.42 (19)
C10—C9—C8	120.83 (16)	C13—O2—H2	109.5
C10—C9—H9	119.6	C15—O3—C18	118.55 (17)
			
O1—C1—C2—N1	50.7 (3)	C13—C14—C15—C16	−1.1 (3)
N1—C3—C4—O1	−49.6 (4)	O3—C15—C16—C17	−179.70 (19)
C10—C5—C6—C7	1.8 (3)	C14—C15—C16—C17	0.6 (3)
N1—C5—C6—C7	−177.56 (19)	C15—C16—C17—C12	0.0 (3)
C5—C6—C7—C8	0.5 (3)	C13—C12—C17—C16	−0.2 (3)
C6—C7—C8—C9	−2.3 (3)	C11—C12—C17—C16	179.64 (19)
C6—C7—C8—N2	−178.64 (18)	C10—C5—N1—C2	171.9 (2)
C7—C8—C9—C10	1.8 (3)	C6—C5—N1—C2	−8.8 (3)
N2—C8—C9—C10	177.85 (17)	C10—C5—N1—C3	25.7 (3)
C8—C9—C10—C5	0.6 (3)	C6—C5—N1—C3	−155.0 (2)
C6—C5—C10—C9	−2.3 (3)	C1—C2—N1—C5	167.9 (2)
N1—C5—C10—C9	177.01 (17)	C1—C2—N1—C3	−44.4 (3)
N2—C11—C12—C17	−175.33 (17)	C4—C3—N1—C5	−168.5 (3)
N2—C11—C12—C13	4.5 (3)	C4—C3—N1—C2	43.8 (4)
C17—C12—C13—O2	179.68 (17)	C12—C11—N2—C8	−177.88 (15)
C11—C12—C13—O2	−0.2 (3)	C7—C8—N2—C11	−154.33 (18)
C17—C12—C13—C14	−0.2 (3)	C9—C8—N2—C11	29.6 (3)
C11—C12—C13—C14	179.93 (16)	C3—C4—O1—C1	54.7 (4)
O2—C13—C14—C15	−179.05 (17)	C2—C1—O1—C4	−55.3 (3)
C12—C13—C14—C15	0.9 (3)	C14—C15—O3—C18	−177.0 (2)
C13—C14—C15—O3	179.24 (16)	C16—C15—O3—C18	3.3 (3)

### Hydrogen-bond geometry (Å, °)

**Table:**

Cg2 and Cg3 are the centroids of the C5–C10 and C12–C17 rings, respectively.

**Table:**

*D*—H···*A*	*D*—H	H···*A*	*D*···*A*	*D*—H···*A*
O2—H2···N2	0.82	1.87	2.600 (2)	148
C14—H14···O3^i^	0.93	2.53	3.412 (3)	158
C18—H18B···O1^ii^	0.96	2.44	3.289 (3)	148
C2—H2A···Cg3^iii^	0.97	2.92	3.799 (4)	152
C16—H16···Cg2^iv^	0.93	2.86	3.663 (3)	146

Symmetry codes: (i) −*x*+3, −*y*+1, −*z*+1; (ii) *x*+3/2, −*y*+1/2, *z*−1/2; (iii) *x*−1, *y*, *z*; (iv) −*x*+5/2, *y*−1/2, −*z*+3/2.
